# The importance of antimicrobial stewardship: An undergraduate perspective

**DOI:** 10.4102/sajid.v39i1.598

**Published:** 2024-07-12

**Authors:** Lodewyk J. de Kock

**Affiliations:** 1 School of Medicine, Faculty of Health Sciences, University of Pretoria, Pretoria, South Africa

**Keywords:** antimicrobial stewardship, antimicrobial resistance, pharmacology, infectious diseases, undergraduate

## Abstract

In this opinion paper, the author reflects on a transformative experience attending an antimicrobial stewardship workshop that shifted their perception of the looming crisis of antimicrobial resistance. The author emphasises the critical need for greater awareness and education at all levels of society, from healthcare professionals to the general population, to truly comprehend the severity of this issue. Drawing parallels between antimicrobial resistance and more recognised threats such as cancer, the author advocates for a multidisciplinary approach and heightened emphasis on education to foster responsible antimicrobial use. They propose practical strategies, including integrating stewardship principles into medical education and advocating for preventive measures such as vaccination. Ultimately, this paper urges individuals to embrace their roles as stewards of antimicrobials and to seize the opportunity to effect change for the betterment of humanity.

**Contribution:**

This opinion paper emphasises the urgent need for greater awareness and education on antimicrobial stewardship. It underscores the importance of multidisciplinary collaboration and proposes practical strategies, particularly in healthcare education. These insights align with the journal’s scope of advancing healthcare education, policy, and addressing global health challenges regarding infectious diseases.

Although it is established that antimicrobial resistance is one of the major crises humankind is currently facing, it is certainly not receiving recognition proportional to the potential it has to wreak absolute havoc.

To be completely transparent, until very recently, antimicrobial resistance and antimicrobial stewardship were not topics to which I had devoted much thought, apart from it being an after-lunch lecture within our curriculum with some very frightening statistics. For example, nearly 5 million deaths were attributed to antimicrobial resistance globally in 2019.^1^ My attitude changed after I had the privilege of attending an antimicrobial stewardship workshop on 26 October 2023 entitled ‘Capacity Building for Antimicrobial Stewardship Champions’, an initiative of the Department of Pharmacology at the University of Pretoria and the School of Pharmacy at Sefako Makgatho University (SMU). My own journey began through a research award to spend time with an academic in research (Prof. N. Schellack, infectious diseases), while attending a symposium hosted by the Tuks Undergraduate Research Forum (TURF) where I was invited to the stewardship workshop. I was then given the opportunity to learn from experts in the field and gain deeper insight by conversing with professionals fighting the good fight daily. Although, to be a medical student among the professionals in attendance was daunting, it was an incredible chance to observe various approaches and ways of critical thinking. Not only did I learn valuable information on the topic, but through observation, I learned how to address these matters head-on. It was ultimately this juxtaposition that inspired my writing. This experience also highlighted a very important principle: *You simply cannot comprehend the severity of a matter which you do not entirely understand*.

I believe this to be the reason why antimicrobial resistance is not on the tip of every tongue and in the headline of every newspaper – so many individuals still have to grasp its true implication. The sad reality is, a large percentage of the population, healthcare workers, students and laypersons alike, do not realise the gravity of the situation.^2,3,4^ Neither do we realise the vitality of active involvement at every level of care in antimicrobial stewardship.

One of the leading causes of death of our time, as is widely recognised, is cancer. Universally the C-word is dreaded and feared; however, research-based predictions state that the death toll because of antimicrobial resistance may surpass that of cancer in the near future, expected to reach 10 million deaths per annum, globally, by 2050.^5^

One important thought, beautifully encapsulated by Margaret Mead can avert this dire situation. She said the following, ‘*Never doubt that a small group of thoughtful, committed citizens can change the world. Indeed, it’s the only thing that ever has*’.^6^ We can contribute to change through passion and commitment by starting to work where we are and with our immediate colleagues.

Her statement highlights the importance of multidisciplinary collaboration and a mindset of continued learning from all team members. It is well known that accountability can affect change.^6^ No individual in the team is more important than the next when it comes to antimicrobial stewardship. Stewardship is a universal issue that requires universal contribution to bring about change.

Another cardinal aspect of antimicrobial stewardship and the war against antimicrobial resistance is an increased focus on the education of not only healthcare students but also the general population. This implies advocating for awareness through educating and encouraging journalists and media publications to appeal to the population at large, as well as equipping healthcare professionals to educate individuals around them with accurate and pivotal information, especially, because acquiring and using antimicrobials without a script is common among our population.^7^ True change starting at home is also an important weapon in our arsenal against antimicrobial resistance. This entails an increased focus on the education of the population about healthy lifestyle choices and behaviour that would ultimately reduce the need for antibiotic use. Appropriate nutrition and improved sanitary and hygiene strategies will reduce the prevalence of infections. Furthermore, the onus of advancing and furthering the stance of our public health rests collectively on all of us.

In addition, I believe that an emphasis on antimicrobial stewardship should be a core component of training all students in healthcare – this includes, but is not limited to, medical students, nursing students, dental students, pharmacy students and all allied students. There is after all no use in understanding which antibiotic to prescribe, when you have no effective antimicrobial agents left at your disposal. Antibiotics are as important a resource as water itself, because without it, the loss of human life is inevitable. This is exactly why we should embody the ethical value of stewardship, aimed at carefully planning for and managing this resource. Education should focus not only on the principles of rational prescribing, such as the careful consideration of drug choice, dosage and duration but also cover a more extensive scope of combatting and preventing antimicrobial resistance. Prevention is, after all, better than cure, especially when there is no cure for antimicrobial resistance. One such preventative strategy, for example, is advocating for and administering vaccines.^8^ The importance of enquiry about whether a patient’s vaccines are up-to-date and patient-education on its benefits should not be overlooked.

Practical strategies to advance antimicrobial stewardship could include curating interest in this field among medical students through increased exposure, reminders and awareness across all aspects of the curriculum, by encouraging the inclusion of contextually relevant information in all lecture materials, and not just limiting it to pharmacology-specific lectures. A multidisciplinary approach to teaching the importance of rational prescription and consideration of resistance across all disciplines would present a unified front, emphasising that each member of the team should pay careful attention to antimicrobial use. Furthermore, involving novel learning strategies, such as interactive case studies, real-world scenario simulation and even gamification could prove effective not only for education but also by teaching critical thinking skills and cultivating a solution-centred approach towards this topic. Another strategy to forward the fight against antimicrobial resistance, and indirectly benefit antimicrobial stewardship, would be to train future clinicians on the consultation-model using the idea that inquiring and advocating for vaccinations is considered core information of a patient’s past medical history.

In conclusion, it is evident that there is still a gap in the awareness, education and general perception of antimicrobial resistance and stewardship, but equally so, there is an opportunity. Fellow students and graduated professionals alike should not miss out on the opportunity to educate themself on the importance of antimicrobial stewardship and the prevention of resistance. At the risk of sounding clichéd, we must realise that we quite literally have the opportunity to change the fate of humankind through the actions we take today.

As a final thought I would like to quote Maya Angelou, who said: ‘*Do the best you can until you know better. Then, when you know better, do better*’.^9^ Let us not be blinded by historical teachings and prejudices, but rather be open to the continued renewal of our convictions, all for the benefit of humanity.

## References

[1] Murray CJ, Ikuta KS, Sharara F, et al. Global burden of bacterial antimicrobial resistance in 2019: A systematic analysis. Lancet. 2022;399(10325):629–655. 10.1016/S0140-6736(21)02724-0 35065702 PMC8841637

[2] Effah CY, Amoah AN, Liu H, et al. A population-base survey on knowledge, attitude and awareness of the general public on antibiotic use and resistance. Antimicrob Resist Infect Control. 2020;9(1):105. 10.1186/s13756-020-00768-9 32653034 PMC7353772

[3] Reddy K, Ramsamy Y, Swe Swe-Han K, et al. Antimicrobial resistance and antimicrobial stewardship in South Africa: A survey of healthcare workers in academic and nonacademic hospitals. Antimicrob Steward Healthc Epidemiol. 2023;3(1):e202. 10.1017/ash.2023.483 38028921 PMC10654946

[4] Leal HF, Mamani C, Quach C, Bédard E. Survey on antimicrobial resistance knowledge and perceptions in university students reveals concerning trends on antibiotic use and procurement. J Assoc Med Microbiol Infect Dis Can. 2022;7(3):220–232. 10.3138/jammi-2022-0008 36337599 PMC9629729

[5] Huang S, Tang Y-W, Cuomo CA, Wang H, Jia X. New threats of antibiotic-resistant bacteria and fungi. Front Med. 2022;9:1078940. 10.3389/fmed.2022.1078940 PMC9716261 36465926

[6] Lutkehaus NC. Margaret Mead: The Making of an American Icon. Princeton, NJ: Princeton University Press; 2018. 10.23943/9780691190273

[7] Sono TM, Yeika E, Cook A, et al. Current rates of purchasing of antibiotics without a prescription across sub-saharan Africa; rationale and potential programmes to reduce inappropriate dispensing and resistance. Expert Rev Anti Infect Ther. 2023;21(10):1025–1055. 10.1080/14787210.2023.2259106 37740561

[8] Meyer HJ. How to build confidence in vaccines. Int J Infect Dis. 2023;130:S48. 10.1016/j.ijid.2023.04.118

[9] McGraw PC. Life strategies: Doing what works, doing what matters. 1st ed. New York, NY: Hyperion Books; 1999.

